# Financial risk of emergency abdominal surgery: a cross sectional study from Ethiopia

**DOI:** 10.1186/s12913-022-08480-7

**Published:** 2022-08-26

**Authors:** Abraham Genetu, Demmelash Gezahegn, Hana Getachew, Andualem Deneke, Abebe Bekele

**Affiliations:** 1 College of Health Sciences, Addis Ababa University, Zambia Street, P.O.Box 8977 Addis Ababa, Ethiopia; 2 St Paul’s Millennium Medical College, Addis Ababa, Ethiopia; 3 University of Global Health Equity, Kigali, Rwanda

**Keywords:** Financial risk, Catastrophic expenditure, Community based health insurance, Global surgery, Access to surgery

## Abstract

**Background:**

The Lancet Commission on Global Surgery suggested six indicators every country should use to measure their surgical systems. One of these indicators, catastrophic expenditure (CE), is defined as money paid for service which amounts to more than 10% of the patient’s total annual expenditure, or more than 40% of annual non-food household expenditure. Ethiopian Ministry of Health has set a target of 100% protection from CE by 2030. However, so far there is lack of studies that assess financial risk of surgery.

**Methods:**

Using a cross sectional study design, financial risk assessment was carried out on 142 patients from Yekatit 12 and Zewditu Memorial hospitals in Addis Ababa, Ethiopia from May 15 to September 15, 2021.

**Results:**

Appendectomy (69.0%), emergency laparotomy (26.1%) and cholecystectomy (4.9%) resulted in mean direct medical expenditures of 111.7USD, 200.70USD and 224.60USD, respectively. Medications and imaging accounted for 60.8 and 13.9% of total treatment cost. By applying the two definitions of catastrophic expenditure, 67.6 and 62.7% of patients sustained CE, respectively Overall rates of CE across procedures were 67.3 and 59.1% for appendectomy, 70.2 and 70.2% for laparotomy, 57.0 and 71.2% for cholecystectomy. Thirty-five (24.6%) patients had some form of insurance, with Community Based Health Insurance being the most common form (57%). Insured patients were less likely to sustain CE with both definitions (AOR 0.09, *p* = 0.002 and AOR 0.10, *p* = 0.006 respectively).

**Conclusion and recommendations:**

Substantial proportion of patients undergoing emergency abdominal surgery sustain CE in Addis Ababa. Medications and imaging take major share of total cost mainly because patients have to acquire them from private set ups. Policy makers should work on availing medications and imaging in public hospitals as well as expand insurance and other forms of surgical care financing to protect patients from CE.

## Background

Access to surgical care is a multidimensional concept that includes availability, geographical accessibility, safety, timeliness, and financial affordability [1]. Safe and affordable surgical and anesthesia care access for all is necessary for human welfare and development [1]. Despite this fact and that surgically amenable diseases comprise a third of all conditions, surgery has been described as the ‘neglected stepchild of global health’ [1, 2]. The Lancet Commission on Global Surgery (LCoGS) estimated that over 5 billion people still lack access to safe and affordable surgical and anesthesia care worldwide [1].

To curb worldwide lack of access to surgery, in 2015 the LCoGS suggested six indicators for country level assessment of surgical systems. Among the six indicators, two are directly related to financial impact of accessing surgery to patients. These two indicators are rate of impoverishing expenditure and catastrophic expenditure (CE) for surgery. Catastrophic health expenditure is defined as the amount of money paid for a health service which amounts to more than 10% of the patient’s annual income or more than 40% of non-food household expenditure [3, 4]. From the 313 million surgeries performed annually worldwide, 33 million people face catastrophic expenditure due to payment for surgery and anesthesia, and an additional 48 million people due to non-medical costs. Most of these patients are from low-income countries [1]. The LCoGS recommended that by 2030, all patients should be protected from impoverishing and catastrophic expenditures due to out-of-pocket payments for surgical and anesthesia care [1].

Following the LCoGS publication in 2015, the Ethiopian Ministry of Health developed a national surgical strategic plan in 2016 (Save Lives through Safe Surgery—SaLTS) with the aim of strengthening the surgical capacity and access. In line with the international recommendations, SaLTS has also suggested assessing the rate of catastrophic expenditure for surgery as one of the indicators for Safe Surgical and Anesthesia Care Programs, and it aims for 100% protection from CE by 2030 [5].

Out of pocket expenditure(OOPE) refers to payments made by the patient to receive health care service [6]. It is the main mode of health care financing in low- and middle-income countries [7]. OOPE has long been the main mode of healthcare financing in Ethiopia while Community Based Health Insurance (CBHI) and insurance from employers are recently being available [8]. In 2017, a report from the World Bank revealed that 97.6% of the Ethiopian population is at risk of impoverishment if they were to undergo surgery. Apart from these estimates, the actual figure of financial risk of surgery is not yet well known.

Fear of financial crisis is one of the most important factors leading to the “First delay” in seeking healthcare [1]. Understanding the economic impacts of surgery on individual patients is essential for the formulation of health policies which promote surgical access. However, few studies have assessed the impact of OOPE on surgical access in Ethiopia. In this study, we assessed financial risk of emergency abdominal surgery on patients from Addis Ababa, Ethiopia.

## Methods and materials

### Study design

This is a cross sectional study done on patients who underwent emergency abdominal surgery at two hospitals in Addis Ababa, Ethiopia. The LCoGS suggested that provision of laparotomy, caesarean delivery, and treatment of open fracture as ‘Bellwethers’ of a system functioning at a level of complexity advanced enough to do most other surgical procedures [1]. This study focused on financial risk of Bellwether procedures because they are indicators for access and that future studies done in lower level and rural hospitals can compare their findings. However, only patients who underwent emergency abdominal surgery were included. Cesarean deliveries were excluded because they are performed free of charge, and patients treated for open fracture were excluded because definitive treatment is not provided in Zewditu Memorial Hospital.

### Study setting

This study was done at two referral hospitals in Addis Ababa, Ethiopia. Zewditu Memorial Hospital (ZMH) is a 280-bed hospital and Yekatit 12 Hospital(Y12H) is a teaching hospital with 350 beds. Their catchment is primarily from residents of Addis Ababa, while sometimes they receive patients from rural hospitals surrounding the city. The hospitals are selected because their catchment is mainly from Addis Ababa residents, and hence they represent a relatively homogenous population.

### Sample size

A similar study from Malawi reported CE of 90% in patients who underwent different types of surgery (11). Using 90% as population proportion, 5% margin of error and confidence interval (CI) of 95%, our sample size is 138.

A review of the monthly audit reports of Yekatit 12 and Zewditu Memorial hospitals from September 2020 to February 2021 showed that the average number of emergency laparotomies including appendectomies were 18 and 17 procedures per month, respectively. Therefore, to include at least 138 patients, data were collected for a period of four months from May 15, 2021 to September 15, 2021.

### Sampling

**Fig. 1:**
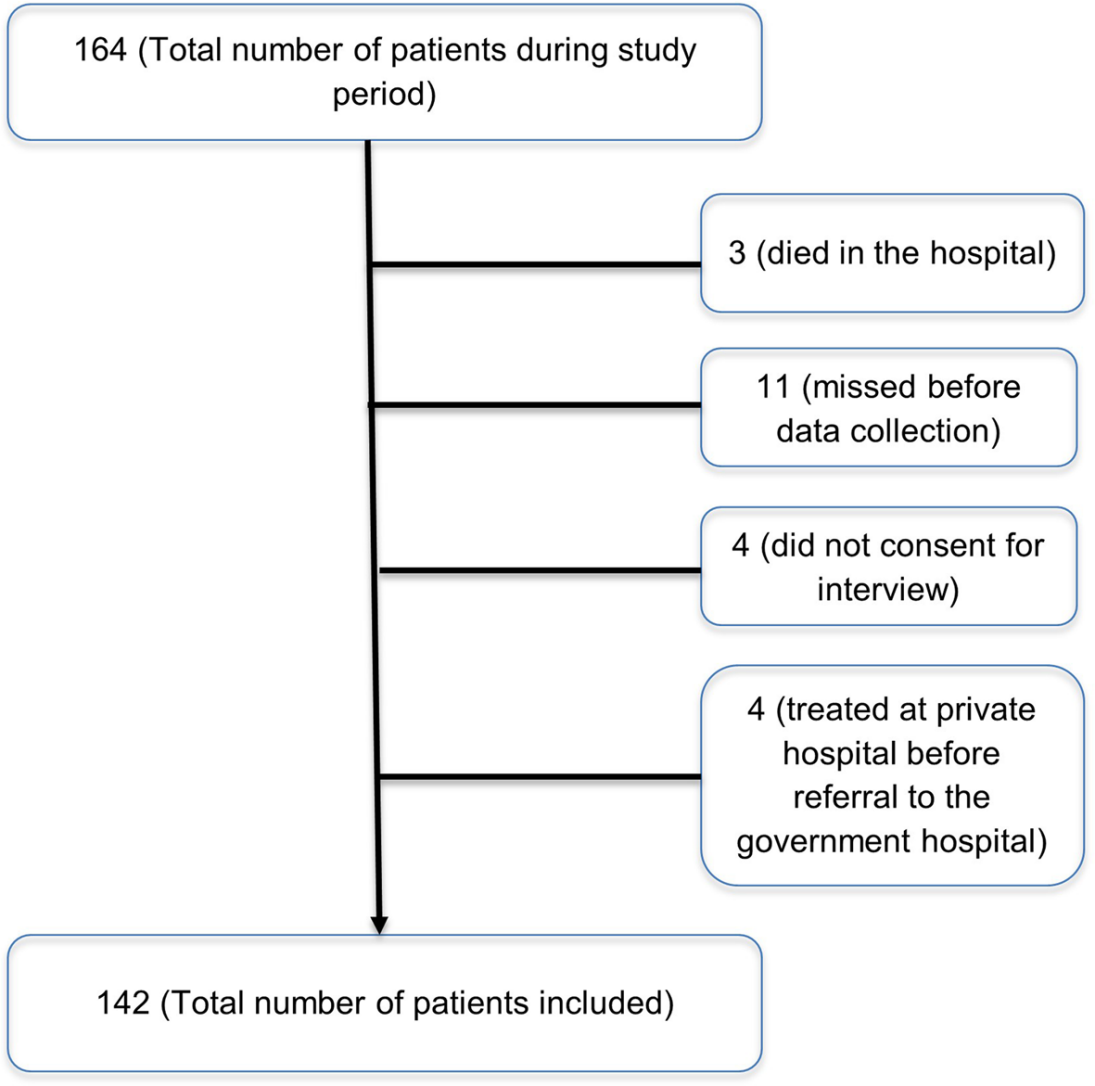
Flow diagram processes of patient inclusion and exclusion

All consecutive patients that meet the inclusion criteria were approached for data collection. During the study period, a total of 164 patients had emergency abdominal surgery (appendectomy, laparotomy, or cholecystectomy). Among these, 142 patients were included in the study. The reasons for exclusion of the remaining 22 patients is shown in the following flow diagram (Fig. 1). Data were collected from both hospitals on all patients during the study period as long as the inclusion criteria were met and there were no preset number of patients required for the hospitals.

### Inclusion and exclusion criteria

#### Inclusion criteria

All adult (> 18 years old) patients who underwent emergency abdominal surgery in the two hospitals during the study period were included.

#### Exclusion criteria

- Age < 18 years old
- Patients who died in the hospital
- Patients treated conservatively for acute abdomen
- Patients who were treated or operated in private hospitals before coming to the study hospitals. These patients were excluded because the amount of payment at the private hospitals were significantly higher than the usual costs at the two public hospitals and the economic status of patients visiting public and private set ups might be different. Therefore, they were excluded to keep relative income homogeneity among patients and to avoid outliers.
- Patients who didn’t consent to participate

### Operational definitions

The operational definitions used for this study are summarized in the following table (Table 1).

**Table 1:** List of operational definitions used for the study

Catastrophic expenditure	A patient is said to have sustained CE when the amount of money paid for the service amounts to more than 10% of the patient’s annual expenditure or more than 40% of non-food household expenditure [9]
Direct medical expense	Any payment related directly to the health care is direct medical expense. These include payments for imaging, laboratory tests, procedures, hospital services [9]
Direct nonmedical expenses	These are payments by patients or attendants to receive the health care like transportation cost, food or similar expenses [9]
Emergency laparotomy	Any procedure done for a patient with an acute abdomen that involves incision of the abdomen to treat or diagnose the condition. This includes, but is not limited to, exploratory laparotomy for trauma, perforated peptic ulcer disease, peritonitis due to complicated appendicitis, intestinal obstructions and other causes of peritonitis. Patients who are operated for appendicitis, through the right lower quadrant incision were separately labeled as Appendectomy
Procedure fee	The amount of money paid for the procedure itself, not including payment to buy anesthesia medications or other materials like normal saline

### Data collection

Data collection was done by trained interns and surgery residents when patients were discharged. A financial risk protection survey tool developed by the Harvard Program in Global Surgery and Social Change and endorsed by the Ethiopian Federal Ministry of Health was used [10]. The tool was validated in the Ethiopian context in Tigray and Amhara Regional States. It was modified to include questions about insurance status and amount of expense covered by insurance. Interview was done with patients and/or care givers.

The data collection tool has questions on sociodemographic data, household size, average monthly income, average monthly expenditure for food and non-food expense categories, and detailed direct medical and nonmedical costs during the index admission.

To avoid recall bias of how much patients paid, data on direct medical expenses were collected from patients’ receipts and by checking from the finance office of the hospitals. Total annual expenditures were collected by interviewing patients on how much they spent on major expenditure categories per month: food, transportation, lodging and other miscellaneous expenses. Monthly expenditures were then categorized in to food expenditure and non-food (non-subsistence) expenditure. Annual expenditures were calculated by multiplying monthly expenditures by 12.

### Determining baseline economic status

Two common ways of measuring baseline economic status are either to measure household per capita expenditure or household per capita income [9]. Using annual income would be more suited for a population with known monthly income [9]. However, most of our patients are self-employed with variable and unpredictable monthly income. Therefore, for this study household per capita expenditure. This method was used by previous studies from low income countries [6, 9, 11, 12]. Patients were interviewed to describe their monthly expenditure in Ethiopian Birr (ETB) and it was converted to United States Dollars (USD) at rate of 1USD = 46.2 ETB according to the rate set by the National Bank of Ethiopia on September 26, 2021.

### Quality assurance

Quality assurance was checked by principal investigators by randomly checking two patients’ data from both hospitals at the end of each week during the study period.

### Data analysis

Data entry, cleaning and analysis were done by SPSS version 25. Expenditures were categorized in three main groups: annual food expenditure, annual nonfood expenditure and total expense of the current treatment. Patients who sustained CE were identified based on the two criteria. Multivariate analysis was used to identify variables that affected risk of sustaining CE. All methods were carried out in accordance with relevant guidelines and regulations.

## Results

### Baseline demographics

Approximately 52% (*n* = 75) patients were treated in Zewditu Memorial Hospital, and the remaining 48% were treated at Yekatit 12 Hospital. All patients were residents of Addis Ababa. Majority of patients were male (54.9) and 69% underwent appendectomy via right lower quadrant incision. Mean duration of hospital stay was 4.7 days (Table 2). Approximately 24% (*n* = 35) of patients had some form of insurance. The most common form of insurance was Community Based Health Insurance (CBHI) (57%), followed by employment insurance schemes and health benefits from charity organizations (Table 2).

**Table 2:** Baseline characteristics of operated patients during the study period

			**Procedures**		
		All patients	Appendectomy	Laparotomy^a^	Cholecystectomy
**Number of patients (%)**		142	98(69.0%)	37(26.1%)	7(4.9%)
**Mean age**		31.0	28.6	35.3	43.6
**Sex**	Male	78 (54.9%)	49	26	3
	Female	64(45.1%)	49	11	4
**Treating hospital**	Yekatit 12 Hospital	67(47.2%)	37	23	7
	Zewditu Hospital	75(52.8%)	61	14	0
**Household size (mean and range)**		3.6 (SD = 3.6)	3.3(SD = 3.7)	4.1(SD = 3.6)	5 (SD = 3.9)
**Length of stay in days**		4.7 (SD = 2.6)	3.2 (SD = 1.6)	7.7 (SD = 4.1)	9.3 (SD = 4.1)
**Insurance status**	Insured	35 (24.6%)	25	7	3
	Noninsured	107 (75.4%)	73	30	4

^a^Laparotomy was done for complicated appendicitis (10), perforated peptic ulcer (9), sigmoid volvulus (7), subphrenic abscess (2), liver abscess (2), tuberculous peritonitis (2), diverticular disease (1), adhesive small bowel obstruction (2), Meckel’s diverticulum (1) and trauma (1) patients

### Hospitalization expenditures

Mean direct medical expenditures for patients who underwent appendectomy, laparotomy and cholecystectomy were 111.7, 200.7 and 224.6 USD. All patients who need emergency laparotomy paid similar amount of procedure fee set by the hospitals, whether the surgery was done for perforated peptic ulcer disease, small bowel volvulus or peritonitis. In this study, the mean cost of procedure accounted for only 2.9% of total expenditure.

**Fig. 2:**
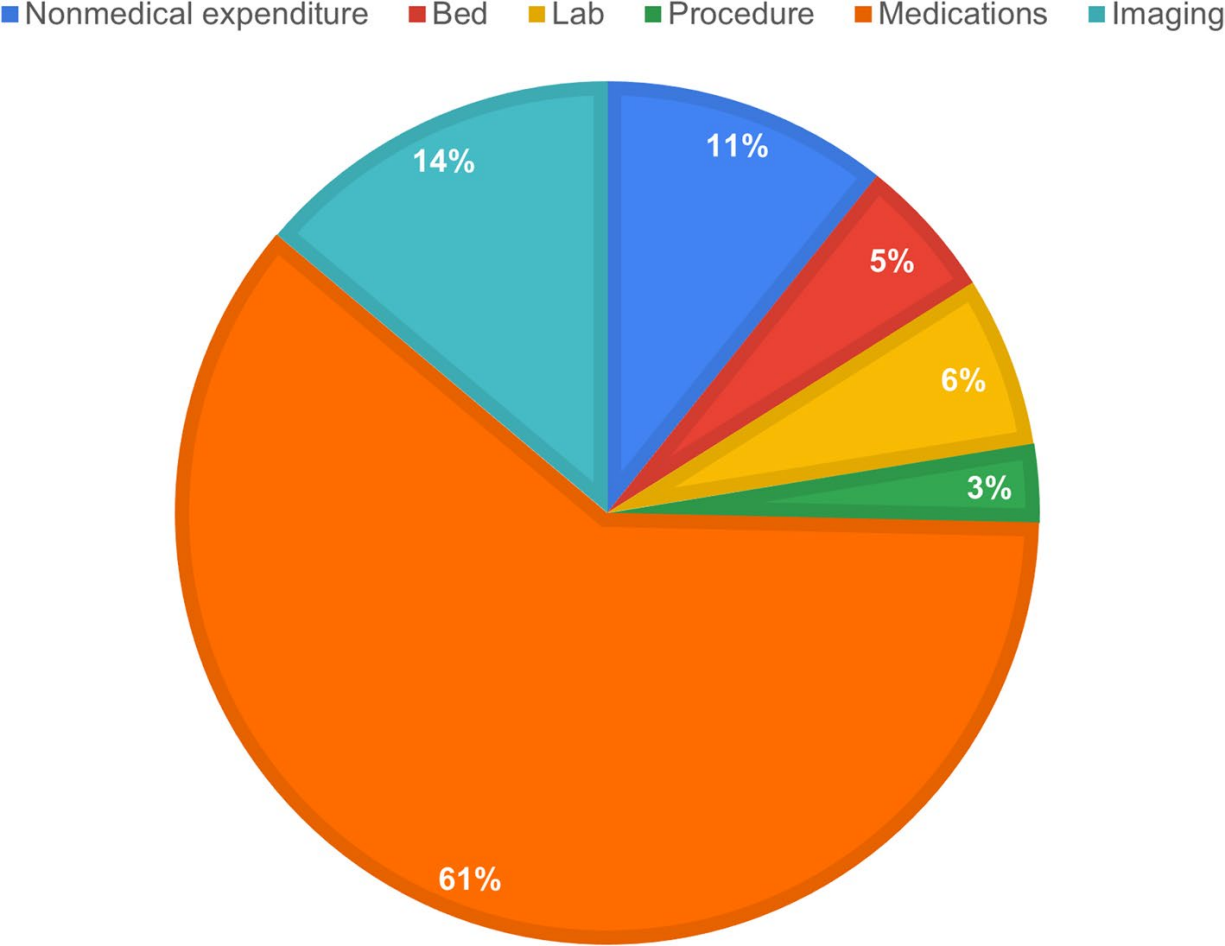
Share of cost of treatment by expenditure category

One way ANOVA test showed significant difference in all mean expenditure categories among the three procedures, except for procedure fee (Table 3). Medications and imaging constituted the major share of direct medical expenditures, accounting for 60.8 and 13.9% of total expenditure respectively (Fig. 2).

**Table 3:** Direct medical and nonmedical expenditure for the three procedures in USD

		Mean Costs in USD (SD)				*P* value
		All patients	Appendectomy	Laparotomy	Cholecystectomy	
**Direct medical expenditures**	Procedure fee	4.5(1.7)	3.9(1.3)	6.4(1.3)	4.8(1.4)	0.601
	Laboratory	10.0(18)	6.4(7.4)	20.2(31.2)	7.3(3.9)	< 0.001
	Imaging	21.8(31.7)	14.6(8.1)	41.2(56.7)	20.3(9.9)	< 0.001
	Medications	95.7(46.3)	80.2(18.0)	1296(47.1)	180.2(125.4)	< 0.001
	Bed and Ward Services	8.4(4.9)	6.7(3.0)	12.3(6.4)	12.1(3.9)	< 0.001
	Total	140.5(*78.2)*	111.7(26.3)	200.7(107)	224.6(141)	< 0.001
**Direct Nonmedical expenditures**		16.9(11.5)	12.8(6.9)	25.6(13.7)	27.9(17.4)	< 0.001
**Total cost (Mean, SD)**		157.4 (86.6)	124.5 (27.6)	226.3 (116.6)	252.4 (158.0)	0.001

### Rates of catastrophic expenditure across the procedures

**Fig. 3:**
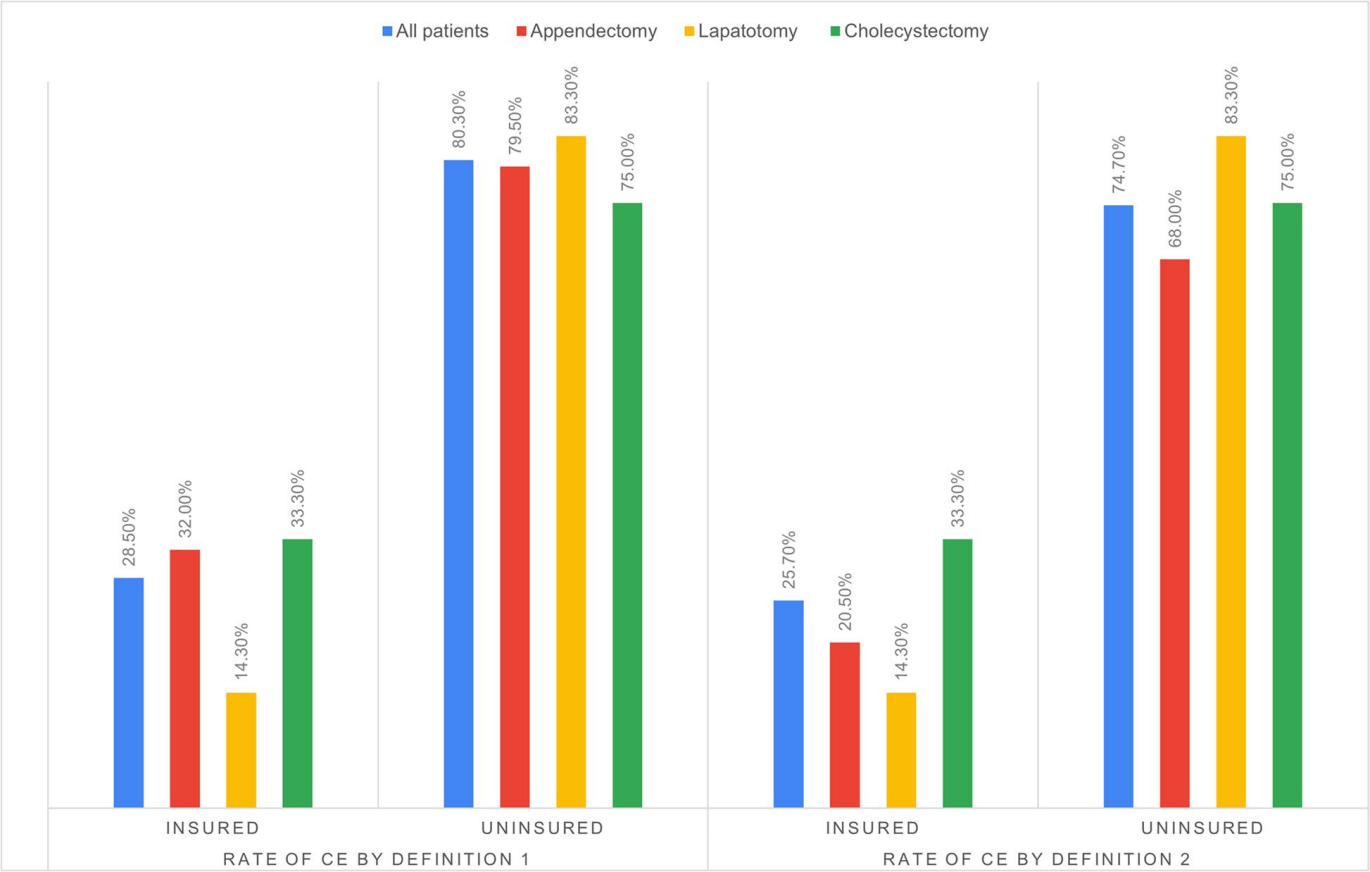
Rates of CE across the procedures in insured and uninsured patients. *Definition 1: Patient is said to have sustained CE when total cost of treatment is > 10% annual household expenditure. *Definition 2: Patient is said to have sustained CE when total cost of treatment is > 40% annual nonfood expenditure

Based on the two definitions for CE, 67.6 and 62.7% of all patients have sustained CE. Overall rates of CE across procedures were 67.3 and 59.1% for appendectomy, 70.2 and 70.2% for laparotomy, 57.0 and 71.2% for cholecystectomy. The differences in CE between these procedures were not statistically significant. Comparison of rates of CE in the three procedures among insured and noninsured patients is as shown below (Fig. 3.) Among all patients, 35 (24.6%) had borrowed money for treatment.

### Insurance schemes and cost coverage by insurance

**Fig. 4:**
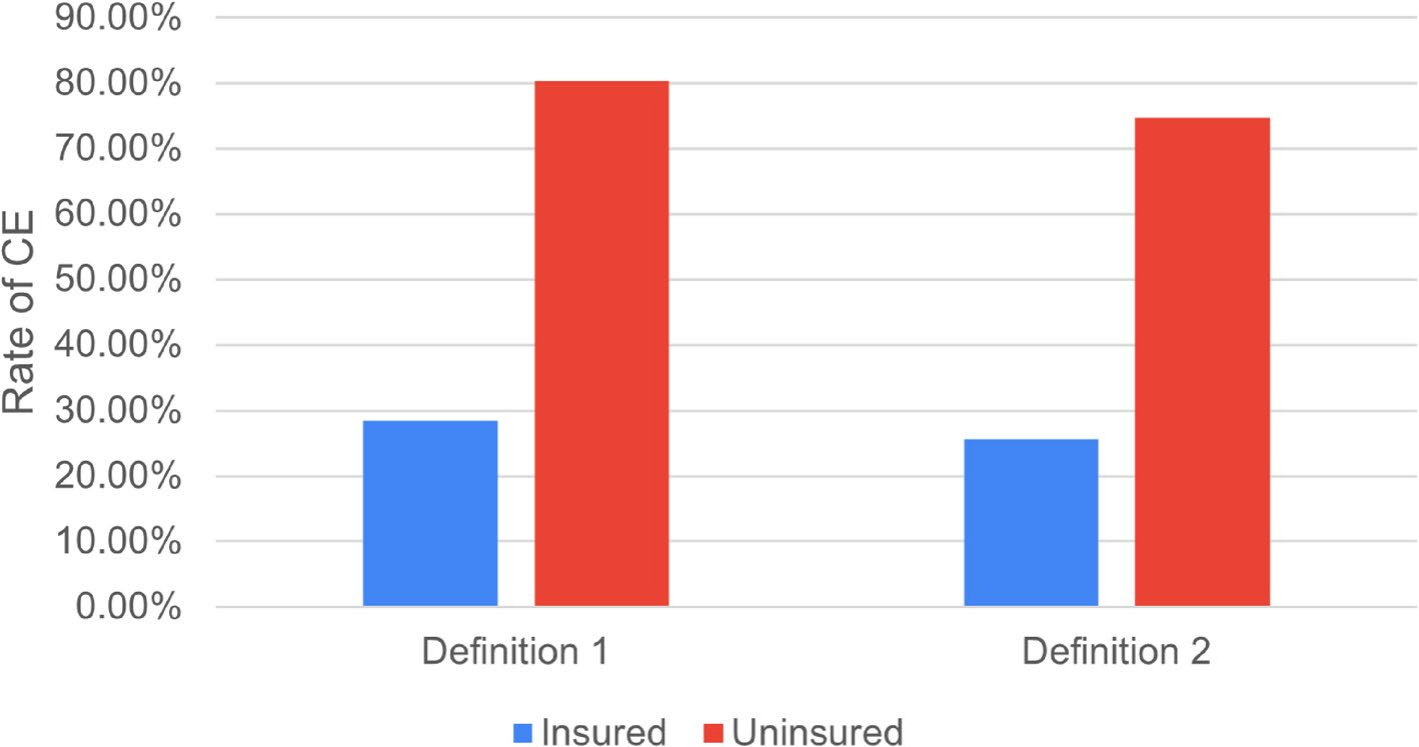
Rates of CE among insured and uninsured patients using both definitions. *Definition 1: Patient is said to have sustained CE when total cost of treatment is > 10% annual household expenditure. *Definition 2: Patient is said to have sustained CE when total cost of treatment is > 40% annual nonfood expenditure

Mean cost of each procedure for patients who have and don’t have insurance is compared in Table 4 below. Patients who have insurance were significantly at lower risk of catastrophic expenditure. When patients with insurance and without insurance were analyzed separately, rate of CE using > 10% annual expenditure is 28.5% in insured patients and 80.3% in uninsured patients (*p* < 0.0001). Similarly, using > 40% annual nonfood expenditure, 25.7% insured patients had sustained CE while 74.7% of uninsured patients sustained CE (*p* = 0.001) as shown below (Fig. 4). If these patients did not have insurance, CE among these groups would be 80.0 and 65.0% using 10 and 40% cut offs.

**Table 4:** Mean total payment for the three procedures for patients with and without insurance in USD

	**Mean Total Expenditure in USD**		
	**Appendectomy**	**Laparotomy**	**Cholecystectomy**
**Insured**	46.89	96.2	89.24
**Uninsured**	124.47	229.93	317.27

### Determinants of CE

Independent variables including the hospital patients were treated in, sex, insurance status, type of procedure, and length of hospital stay were analyzed using binary logistic regression. Among these, the presence of insurance was the only variable with significant association in protecting patients from CE (Table 5).

**Table 5:** Logistic regression of independent variables to assess determinants of CE

	Catastrophic Expenditure = OOPE > 10% of annual total household expenditure			Catastrophic Expenditure = OOPE > 40% of annual non-food expenditure		
Variables	Adjusted Odds Ratio (aOR)	95% C.I	*p* value	Adjusted Odds Ratio (aOR)	95% C.I	*p* value
Sex (Male)	1.047	(0.453, 2.419)	0.914	1.890	(0.85, 4.205)	0.119
Procedure	0.999	(0.993, 1.005)	0.748	0.998	(0.992, 1.003)	0.399
Length of stay	0.913	(0.798, 1.046)	0.189	0.923	(0.809, 1.052)	0.23
Household number	1.179	(0.935, 1.486)	0.163	0.900	(0.720, 1.125)	0.354
Any Health Insurance	0.091	(0.037, 0.226)	0.002	0.101	(0.040, 0.255)	0.006

## Discussion

Our study showed that about two third of patients undergoing emergency abdominal surgery sustained CE. Access to surgical care is a multidimensional concept that includes availability, geographical accessibility, safety, timeliness, and financial affordability [1]. In this regard, even though surgical care is physically available, it is still unaffordable for many patients in the capital city. To increase access to surgery throughout the world, protection of all surgical patients from CE resulting from accessing surgical care is one of the recommendations made by the LCoGS. This study’s findings show that there is still work to be done to make essential surgery accessible.

The LCoGS estimates that 81.2 million people endure CE from accessing surgical care every year. However, these estimates do not consider patients who are not able to access surgical and anesthesia care in the first place. Globally, about half of the population are at risk of CE if they have to need surgery [1, 7]. Estimates of rate of catastrophic expenditure for surgical care in Sub Saharan Africa is highly variable across countries, the highest being 91% for Burundi [13]. Apart from modeling studies and nationwide estimates, studied on surgical patients undergoing specific procedures in Sub Saharan African countries have shown significant risk of CE. Study from Malawi showed that out of 137 patients who underwent hernia surgery in central and district hospitals, 90 to 97% of patients sustained CE [12]. This was higher to magnitude of CE from our patients (62.7 to 67.6%), which can be explained by the fact that the study from Malawi added opportunity costs due to income forgone because of hospital admission for surgery as indirect expenditure. They found that such costs often exceeded the direct medical costs [12]. Another study from Rwanda showed that 28% patients were at risk of catastrophic health expenditure from surgery for peritonitis. Their patients were different from the study in Malawi by the fact that about 98% of their patients had CBHI which explains lower risk of CE. Compared to our study, the rate of CE was similar to patients who had insurance [11]. In similar study from Uganda where surgical care is free in government hospitals, 31% of patients faced a catastrophic expenditure from elective and emergency procedures [9]. The main reasons their patients incurred CE were unavailability of drugs and diagnostic tests in government set ups.

Despite the low cost of procedures and ward services, many of our patients incurred CE due to diagnostics and medications. Key cost drivers accounting for more than 70% of the total cost of treatment of our patients included payment for medications and imaging. In our study, these costs were driven up because they were not available within the care facilities and involved external referral to private facilities. For example, an abdominal ultrasound costs 53ETB in Yekatit 12 Hospital, while it costs from 500 to 900ETB in private diagnostic centers as was noticed during data collection. Unavailability of these services was also the main factor for patients to sustain CE in a study from Uganda, where surgical service is provided for free [9]. Therefore, provision of quality diagnostic service and basic medications at government hospitals could significantly alleviate the burden of CE.

### CBHI and other insurance schemes in Ethiopia

Attempts to address a long history of underfinancing of Ethiopian healthcare led to development of health care financing strategy in 1998 [14]. The strategy suggested health insurance as a tool to generate more revenue and increase health service utilization. The Federal Ministry of Health of Ethiopia identified Social Health Insurance (SHI) for the formal sector employees and Community Based Health Insurance (CHBI) for the informal sector to be utilized as insurance schemes. However, SHI didn’t get implemented, while in 2010, pilot studies in some rural Ethiopia implementing CBHI showed promising results [15]. Based on such findings, in 2011, CBHI was launched to wider parts of the country. The scheme would set premiums of 350ETB per household that will finally cover all healthcare expenses in public facilities [8]. In 2017, CBHI was implemented in Addis Ababa under regulation number 86/2017 [16]. The CBHI covers all medical expenses in any government hospital, except long term dialysis and sight glass purchase, however, CBHI subscribers can only be those working in the informal sector. Families who are indigent can subscribe to the CBHI for free and still get the same service as those who are paying. Apart from CBHI, some employers provide a variable degree of health insurance ranging from payment for medication and procedures, to all costs including transportation and lodging.

In our study, having insurance was the only independent variable associated with significantly lower risk of sustaining CE. This is consistent with findings from similar study in Rwanda [11]. Rate of CE using > 10% annual expenditure is 28.5% in patients who have insurance and 80.3% in noninsured patients (*p* < 0.0001). Similarly, using the other criteria (> 40% annual nonfood expenditure), 25.7% patients with insurance had sustained CE while 74.7% of noninsured patients sustained CE (*p* < 0.0001)*.* If these patients did not have insurance, CE among these groups would have been 80.0 and 65% using 10 and 40% cut offs, making the rate of CE comparable to noninsured patients. Although having insurance was significantly associated with lower CE in our patients, it did not absolutely protect all patients from financial risk of surgery. This was mainly because many medications and basic imaging like ultrasound and CT scan were not available or accessible at the treating hospitals as was in similar study from Uganda [9]. Provision of these services could help protect from CE for both insured and noninsured patients.

Overall, with only 8 years left from 2030, by which time LCoGS and SaLTS recommend to protect all surgical patients from CE, our results suggest that more work is necessary within the Ethiopian health system to achieve that goal.

## Conclusion and recommendations

The financial burden of having emergency abdominal surgery is high in Addis Ababa, Ethiopia. About two-third of our patients endure CE from such procedures. Having insurance is shown to decrease risk of CE significantly. However, less than one third of our patients are under health insurance cover, and CBHI is still limited to the indigent population. Policy makers should work on expanding access to insurance schemes for larger proportions of the population.

Service providers and stakeholders should also make sure medications and imaging are available at government institutions to alleviate financial burden of surgical patients. Despite having insurance, up to a third of patients still experience CE from surgery because of lack of these services at the government hospitals. The burden is even greater for patients who are uninsured. Therefore, ensuring that necessary medications and basic imaging (particularly ultrasound and computed tomography scans) are critical to decrease risk of CE.

## Limitations of this study

Since our study was done on patients who already underwent surgery, it could fall short to predict the full estimate of the population that would be at risk of CE if they were to undergo surgery despite their affordability status. In addition, although our findings could be projected to regional cities; they cannot depict the exact picture of financial risk of surgery for patients residing in rural areas.

This study enrolled patients undergoing emergency surgery which didn’t require longer duration of stay and extensive surgery. Therefore, further study should be done to assess the financial risk of other surgeries like Whipple’s procedure or chest procedures that would require more diagnostic tests, subspecialty expertise and longer hospital stay.

Findings of this study should be interpreted keeping in mind that calculations of food and non-food household expenditure depended on recall based on predetermined expense categories. This raises the possibility of inaccurate reporting of expenditures or missed expenses which do not align with a category. In addition, indirect medical expenses that could have happened to patients because of productivity loss or loss of salary during the treatment are not included in assessing rates of CE. This might undermine the number of patients who sustained CE.

## Data Availability

The datasets used and/or analyzed during the current study are available from the corresponding author on reasonable request.

## Abbreviations

CBHI: Community Based Health Insurance
CE: Catastrophic Expenditure
ETB: Ethiopian Birr
LCoGS: Lancet Comission on Global Surgery
SaLTS: Saving Lives thru Safe Surgery
USD: United States Dollars
